# Development and physicochemical characterization of novel porous phosphate glass bone graft substitute and in vitro comparison with xenograft

**DOI:** 10.1007/s10856-021-06532-8

**Published:** 2021-05-17

**Authors:** Niketa Chauhan, Nilay Lakhkar, Amol Chaudhari

**Affiliations:** SynThera Biomedical Pvt. Ltd. 100, NCL Innovation Park, Dr. Homi Bhabha Road, Pashan, Pune, Maharashtra 411008 India

## Abstract

The process of bone regeneration in bone grafting procedures is greatly influenced by the physicochemical properties of the bone graft substitute. In this study, porous phosphate glass (PPG) morsels were developed and their physicochemical properties such as degradation, crystallinity, organic content, surface topography, particle size and porosity were evaluated using various analytical methods. The in vitro cytotoxicity of the PPG morsels was assessed and the interaction of the PPG morsels with Dental Pulp Stem Cells (DPSCs) was studied by measuring cell proliferation and cell penetration depth. The cell-material interactions between PPG morsels and a commercially available xenograft (XG) were compared. The PPG morsels were observed to be amorphous, biocompatible and highly porous (porosity = 58.45%). From in vitro experiments, PPG morsels were observed to be non-cytotoxic and showed better cell proliferation. The internal surface of PPG was easily accessible to the cells compared to XG.

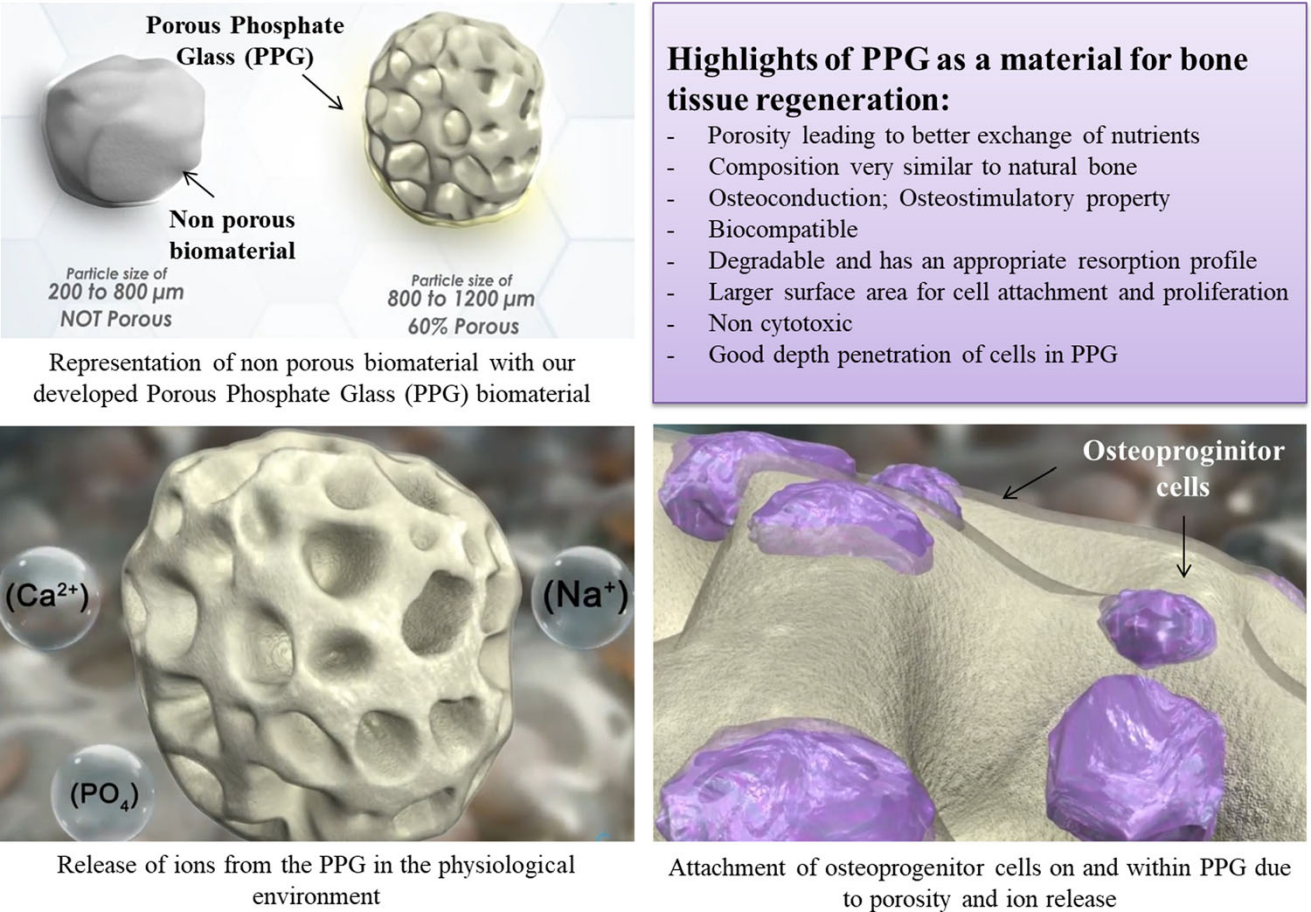

## Introduction

Over the years, the role played by biomaterials in hard tissue regeneration applications has evolved from serving simply as an inert support to actively initiating and facilitating a targeted tissue response through mechanical and physicochemical cues [1]. Ideal bone grafts are void filler materials that induce bone formation by acting as a mineral reservoir and thus promoting wound healing [2]. Bone grafting is possible because bone tissue has the ability to regenerate. Therefore, bone grafts are an active area of research wherein substrates or scaffolds made from different materials are tested for their use in tissue engineering and regenerative therapy [3]. Osteoconduction, osteoinduction, and osteogenesis are the essential characteristics of bone grafts required for bone regeneration [4, 5]. Osteoconduction allows cellular infiltration and vascularization through cell attachment to the external and internal surfaces of a porous graft. [1, 6]. Osteoinduction implies recruitment of mesenchymal cells and the stimulation of these cells to differentiate into the osteogenic lineage [1]. Osteogenesis refers to the osteo-differentiation of cells which eventually leads to formation of new bone matrix [7]. Bone grafting is a surgical procedure frequently performed in oral, maxillofacial and orthopedic surgeries to treat patients with missing or damaged bone tissue. In dentistry, one of the most common uses of bone grafting is to re-establish the edentulous area of a missing tooth before placement of an implant. For example, bone grafting can be helpful where ridge defects are developed as a result of surgery, trauma, infection, congenital malformations, or where pneumatization of maxillary sinus has occurred after tooth loss. With technological advancements, bone grafting has significantly shifted from autologous grafts (autografts) to allogeneic grafts (allografts) to alloplastic grafts (alloplasts) [2]. Alloplasts are synthetically produced and their advantages over autografts and allografts include easier availability, easier scale-up, longer shelf-life etc. Also, there is no risk of disease transmission and infection, justifying a significant shift in use of alloplasts [8–10]. Research worldwide into these grafts is at present geared towards continuous optimization of characteristics such as crystallinity, glass transition temperature, solubility etc. to fulfill the clinical requirements of bone grafting materials [7].

Bioactive glasses are calcium and phosphate-containing synthetic biomaterials, which are inherent components of the bone [11, 12]. Bioactive glasses have several unique properties in comparison with other synthetic bone graft substitutes. They are demonstrated to be more reactive than inert materials like hydroxyapatite (HA) and beta-tricalcium phosphate (TCP). The intrinsic properties of bioactive glass, particularly the release of physiologically beneficial ions, give them the ability to promote natural bone regeneration and bone-bonding ability [13]. Also, depending on the chemical composition, they have controllable rates of resorption and bioactivity [12]. The release of ions stimulates cell proliferation and differentiation into the osteogenic lineage. The glass is fully resorbed and absorbed over a period of time and replaced by bone [11, 14]. There are three main types of bioactive glasses distinguished by the glass formers used and called as silicates, borates and phosphate glasses. Silicate bioactive glass, commercialized as 45S5 Bioglass^®^, has its main application in hard tissue repair [15]. Borate-based glasses produce a more rapid conversion of the glass to hydroxyapatite than silicate glass, which can be helpful when using these glasses as scaffold materials for bone tissue engineering [16]. But silicate and borate glasses have limited compositional range due to which they do not suit many applications. Phosphate-based glasses can offer enhanced properties due to the possibilities for modification in the composition in order to elicit a particular biological response [15].

In recent years, phosphate glasses have been extensively studied for a variety of biomedical applications as possible alloplasts for two key reasons. Firstly, the degradation of phosphate glasses is highly controllable in various solubilizing media. For example, modification of ternary glasses such as P_2_O_5_–Na_2_O–CaO by the addition of suitable metal oxides to form quaternary or higher-order systems can reduce glass solubility by several orders of magnitude [17, 18]. Metal oxides are the most efficient way of regulating the rate of degradation, since their addition increases the covalent bonds within the glass structure. Also, metal ions released from these glasses may exercise biological functions such as antimicrobial effects and/or positive effects on cell proliferation and tissue regeneration [13, 19]. Secondly, the main elemental constituents of phosphate glasses (e.g., Na, Ca and P) are found in the inorganic mineral phase of bone [20]. When implanted in living tissue, phosphate glass materials undergo changes in structure and chemical composition, resulting in an osteoconductive and osteostimulative effect [12, 21]. Such degradation of phosphate glasses has also been shown to be hemostatic. The released Ca ions concentrate the blood constituents and support formation of blood clots [22]. Quaternary P_2_O_5_–Na_2_O–CaO–TiO_2_ glass containing 5% TiO_2_ has a major impact on the ability of the glass surface to provide a stable substrate for cells to attach, proliferate and, in the case of stem cells, to differentiate along osteogenic or chondrogenic pathways [23]. Researchers are actively focusing on titanium phosphate glasses due to their highly favorable material properties and ability to elicit a positive response from bone tissue [17, 24–26]. It is worth noting that along with the material physico-chemical properties, porosity and interconnectivity also play significant roles in determining graft performance and overall success of the bone graft substitute [27]. Incorporation of porosity within the graft may provide an approach for increasing the success of graft vascularization, along with an interconnected pore structure which allows high permeability for fluid transport and nutrient exchange. The pores are also important for bone tissue formation as they allow proliferation and migration of osteoprogenitor cells, inducing adequate osteoconduction and eventually playing a role in osteogenesis [28–30].

On the basis of the existing literature, a novel bone graft substitute named Porous Phosphate Glass (PPG) was developed. The composition of the titanium phosphate glass used for making PPG has been shown to bond to both hard as well as soft tissue [31]. The major advantage of PPG is its porosity leading to the possibility of better exchange of nutrients and also providing a larger surface area for cell attachment and proliferation. In this study, extensive physicochemical characterization of PPG morsels was carried out. Also, in vitro studies were carried out to determine cytotoxicity and interaction of PPG with dental pulp stem cells (DPSCs) in comparison with a commercially available xenograft (XG) material [8, 32, 33] as a positive control.

## Materials and methods

### Materials

The following chemicals were used for the production of PPG (all the chemicals used were of LR grade): phosphorus pentoxide (P_2_O_5_), calcium carbonate (CaCO_3_), N N- methylene bisacrylamide, Triton X100, ammonium persulfate and N, N, N′, N′- tetramethyl ethylenediamine (all from Loba, Mumbai, India); sodium carbonate (Na_2_CO_3_, Merck, Darmstadt, Germany); titanium oxide (TiO_2_, Sigma-Aldrich, Saint Louis, MO, USA); methacrylamide (TCI, Tokyo, Japan). Ammonium polyacrylate was kindly gifted by BASF, Mumbai, India.

### Methods

#### Glass making

The glass was made with a composition of 50% P_2_O_5_, 40% CaO, 5% Na_2_O and 5% TiO_2_. All composition values are in Mol%. The required amounts of precursors were weighed in a Platinum-Rhodium (10%) crucible (Manilal Maganlal and Company, Mumbai, India) and mixed thoroughly using a flat spatula. The crucible was then placed in an Ants TempSys BLF 1600 raising hearth furnace (Ants Ceramics, Mumbai, India). The mixture in the crucible was melted at 1300 °C for 1 h and then quenched on a steel plate. The phosphate glass thus formed was milled using a planetary ball mill (Insmart, Hyderabad, India). This glass powder was further used for foam making.

#### PPG morsel making (in-situ polymerization)

PPG samples were made as per the procedure described in WIPO publication number WO/2018/167724. Appropriate quantities of glass powder; water; methacrylamide; N N′ methylene bisacrylamide; ammonium polyacrylate and Triton X100 were mixed vigorously using an overhead mechanical stirrer (Ika, Staufen, Germany). A foamy liquid was formed to which ammonium persulfate and N, N, N′,N′- Tetramethyl ethylenediamine were added, and the liquid was stirred until gelling. The gel thus formed was cut into smaller blocks with approximate dimensions of 1 × 1 × 1 cm. The blocks were sintered by heating them to 400 °C for 1 h. Then, final sintering was carried out at temperatures of 700, 650, 625, 600 and 575 °C for 1 h. These different temperatures were used for the last step of sintering to understand the effect of sintering temperature on the crystalline properties of the sintered foam. The sintered foam was manually crushed using a mortar and pestle followed by sieving using a sieve shaker (Biotechnics India, Mumbai, India) for 40 min. Sieves (Jayant Scientific, Mumbai, India) were selected such that two fractions of morsels of mesh sizes between 210 µm (BSS 72) & 420 µm (BSS 36) and 420 µm (BSS 36) & 850 µm (BSS 18) were obtained.

### Characterization

#### X-ray diffraction (XRD)

To determine the presence of crystallinity [34], XRD was performed. A comparison of the XRD spectra of the glass and the morsels after sintering was carried out. For this experiment, the XRD spectra of original glass powder (used for making the morsels) and powders of foam sintered at 700, 650, 625, 600 and 575 °C were obtained. The XRD measurements were carried out in transmission mode on a D8 Advance X-ray Diffractometer (Bruker, Germany) with Cu Kα radiation and λ = 1.54 Å. The XRD data were collected at 2Ө values in the range 10–70°.

#### Thermo-gravimetric analysis (TGA)

To further confirm the crystallization temperature, TGA was carried out on a glass powder sample and the data obtained was converted to Differential Thermal Analysis (DTA) by plotting the heat flow against the temperature. The measurement was carried out using STA 6000 device and the data was captured using Pyris Thermal Analysis Manager Software (PerkinElmer, MA, USA). The heating program used was from room temperature to 900 °C at a heating rate of 20 °C/min in air. The parameters measured were glass transition temperature (T_g_), onset of crystallization (T_c(ON)_), crystallization temperature (T_c_) and melting temperature (T_m_).

#### Fourier transform infra-red (FTIR)

To check the presence of organic matter in the sintered samples, FTIR Spectroscopy was employed. The FTIR spectra were recorded on Bruker alpha instrument (Bruker, Germany) with OPUS 7 software. Glass samples (glass and the morsels after sintering in powder form) were evaluated and infra-red spectra were generated in the mid-infrared region of 3000–400 cm^−1^. Spectra were obtained at 4 cm^−1^ resolution averaging 128 scans.

#### Scanning electron microscopy (SEM)

Two fractions of morsels were evaluated (Refer–“**PPG Morsel Making** (**In-Situ Polymerization)”** section). PPG morsels were mounted onto carbon adhesive discs attached to aluminum SEM specimen stubs. The stubs were then sputter-coated with gold by means of a Polaron E5100 coating device (Polaron CVT, Milton Keynes, UK). The coated samples were observed with a JSM 5410LV scanning electron microscope (JEOL, Japan) at an operating voltage of 20 kV. The images were taken at 500X magnification.

#### Particle size distribution (PSD)

The exact particle size distribution of the morsel fraction of mesh size 420 (BSS 36)–850 µm (BSS18) was evaluated by laser diffraction using a Malvern Mastersizer (Malvern Instruments, UK). The samples were measured by the dry dispersion method. The following parameters were used: particle refractive index of 1.52, particle absorption index of 0.100 and laser obscuration of 1.91%. The PSD was determined by the distribution of scattered light energy by Mie scattering model.

#### Mercury porosimetry

The porosity and pore size distribution of PPG was obtained by mercury intrusion [35] using a AutoPore IV 9500 V1.09 device (Micromeritics Instrument Corp., GA, USA) in the pressure range between 0.05 and 2000 atm, corresponding to a range of pore diameters between 400 µm and 0.006 µm, respectively. From the pressure versus intrusion data, pore size distribution and porosity of PPG were determined.

#### Density

The bulk density of PPG morsels was measured and compared with that of the original glass. Density measurements were carried out on the basis of Archimedes’ principle using triplicate samples of glass fragments. An analytical balance (Mettler Toledo, UK) with a density measurement attachment was used for this purpose. Absolute ethanol was used instead of water as the immersion liquid since the glasses are water-soluble in nature. The following formula was used to calculate the glass density:

$$\rho _{{\mathrm{glass}}} = \left[ {M_{{\mathrm{air}}}/\left( {M_{{\mathrm{air}}} - M_{{\mathrm{ethanol}}}} \right)} \right] \times \rho _{{\mathrm{ethanol}}}$$

Where *M*_*air*_ and *M*_*ethanol*_ are the masses of the sample in air and ethanol, respectively, and *ρ*_*ethanol*_ is the density of ethanol at ambient temperature (g.cm^−3^).

#### Degradation study

The study was carried out with PPG morsels at five time points: Day 1, 4, 7, 11 and 14 (*n* = 5 for each time point). PPG morsels (200 mg) were mixed with 10 mL of deionized water in a closed container. At each time point, water from the samples was removed and the remaining PPG morsels were dried in hot air oven (Biotechnics India, Mumbai, India) overnight at 70 °C. After drying, the samples were weighed to determine the weight loss as compared to the weight before start of the experiment. The data is represented as a plot of cumulative weight loss vs. time.

### In vitro studies

#### Cytotoxicity

The test was performed in accordance with ISO 10993-5:2009. For the test, extracts of PPG were prepared in Eagle minimum essential medium (EMEM) at 37 °C for 24 h. L929 cells were seeded in a 96-well plate at a density of 10,000 cells per well, treated with various concentrations of extracts (100, 50, 25, 12.5 and 6.25%) and stored at 37 °C in a 5% CO_2_ incubator for a period of 24 h. Complete media was used as the negative control and 1% phenol in complete media was used as the positive control. After the incubation period, MTT assay was conducted to assess the cytotoxicity. For MTT, extract containing media was removed, 30 µL of MTT (1 mg/mL in EMEM) reagent was added to each well, and the plates were incubated for 3 h at 37 °C. The MTT containing media was removed and 100 μL DMSO was added to each well. After 10 min of mild mechanical shaking, the optical density (OD) was measured for each well at 570 nm keeping 600 nm as a reference wavelength in a plate reader (synergy 4 multimode microplate reader-BioTEK, VT, USA). The percentage cell viability was calculated using the following equation:

$${\mathrm{\% }}\,{\mathrm{Cell}}\,{\mathrm{Viability = }}\left( {{\mathrm{OD}}\,{\mathrm{Test/OD}}\,{\mathrm{Control}}} \right) \times {\mathrm{100}}$$

A material is considered as cytotoxic if the cell viability is <70% and non-cytotoxic if the cell viability is >70%.

#### SEM of DPSCs on particulate biomaterial

DPSCs were seeded on to 200 mg PPG morsels at a seeding density of 100,000 cells per well in a 24 well plate. At day 14, to fix the cells for SEM imaging, the medium in the wells was replaced with 3% glutaraldehyde (Loba, Mumbai, India) in 0.14 M sodium cacodylate (Otto, Mumbai, India) buffer. The morsels with fixed cells were kept at 4 °C overnight. Glutaraldehyde was removed and the samples were treated with a graded series of ethanol (Merck, Darmstadt, Germany) with increasing concentrations of 50, 70 and 90% for 10 min. The specimens were then treated with 100% ethanol for 30 min and this procedure was repeated for three times. The 100% ethanol was replaced with hexamethyldisilazane (SRL, Mumbai, India) for 2 min. After removing hexamethyldisilazane, the plate was left to dry overnight in a desiccator. The images were taken at different magnifications 500X, 1000X, 2000X and 4000X with a JEOL JSM-6360 scanning electron microscope (JEOL, Japan).

#### Cell proliferation

PPG morsels with mesh size fraction of 420–850 µm and a commercially available XG commonly used in dental surgeries for bone regeneration (Bio-Oss^®^, Geistlich Biomaterials, Wolhusen, Switzerland), were used in a comparative cell proliferation study. PPG was heat sterilized (at 180 °C for 3 h in hot air oven). XG was used as received from the manufacturer. Low attachment 24 well tissue culture suspension plates (Thermo scientific-Nunc, Cat #144530) were used. Polystyrene 24 well cell culture treated plates (Thermo scientific-Nunc, Cat #142475) were used as the positive control. Biomaterial samples of 200 mg were added per well. Each test sample was prepared in triplicate. Each well (Test and Control) received 100,000 cells via 1 mL medium. Plates were incubated at 37 °C with 5% CO_2_ and more than 90% humidity during the experiment. Alamar Blue assay (EZBlue^TM^ Cell Assay Kit, Himedia, Mumbai, India) was carried out after 1, 7, 14, 21 and 28 days of incubation. EZBlue reagent was added (10% of the total culture volume per well). The plates were incubated in the dark at 37 °C in a 5% CO_2_ environment for 4 h. Absorbance measurements were carried out at 570 nm keeping 600 nm as a reference wavelength on a multimode microplate reader (TecanInfinite 200 pro, Tecan Switzerland).

#### Confocal microscopy

Comparative confocal microscopy of DPSCs seeded on to PPG morsels and XG at a seeding density of 100,000 cells per well was carried out. Cell staining was carried out using Image-iT™ LIVE Intracellular Membrane and Nuclear Labeling Kit (Invitrogen, Oregon, USA). CellTrace™ BODIPY^®^ TR methyl ester (Excitation and emission maxima 598/625 nm) was used to stain the intracellular membrane and Hoechst 33342 dye (excitation/emission maxima 350/461 nm) was used to stain the nucleic acid. Stained samples were incubated for 10 min at 37 °C. Once the labeling was complete, the staining solution was removed; later the samples containing cells were washed twice in cell-culture medium, and were maintained as-is for 5 min in cell-culture medium at 37 °C. The samples were then viewed using standard filter sets in a Leica TCS SP8 Spectral Confocal Laser Scanning Microscope with LAS X imaging software (Leica Microsystems, Germany).

### Statistical analyses

For the comparison of cell proliferation in presence of PPG and XG, statistical analysis was performed using Microsoft Excel (Microsoft Corporation; Microsoft Office Standard 2010) via two tailed, homoscedastic t-test. A 98% confidence level was considered significant (*p* < 0.02).

## Results

### Characterization

#### XRD

**Fig. 1:**
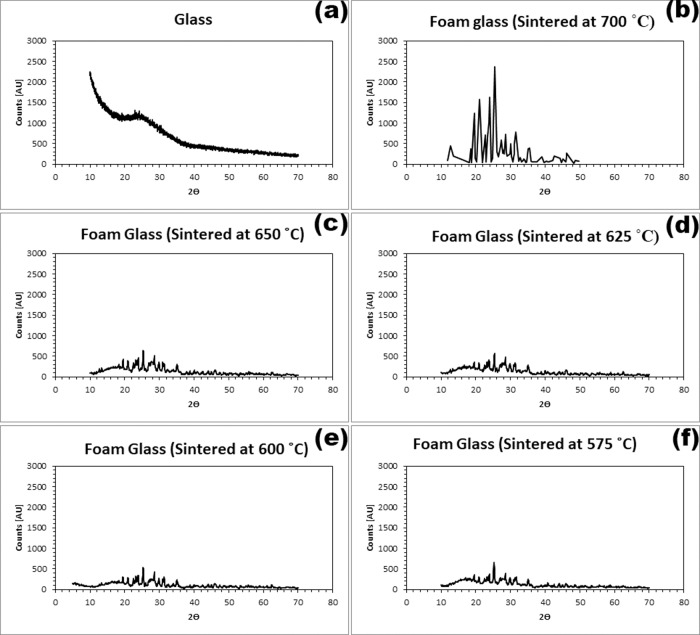
Effect of sintering temperature on crystallinity of PPG morsels. **a** XRD spectrum of the glass before sintering the morsels; XRD spectra of PPG morsel powder after sintering at (**b**) 700 °C, (**c**) 650 °C, (**d**) 625 °C, (**e**) 600 °C and (**f**) 575 °C

Figure 1 summarizes the XRD spectra of the original glass (Fig. 1a) in comparison with the morsels sintered at 700, 650, 625, 600 and 575 °C (Fig. 1b–f). The glass sintered at 700 °C (Fig. 1b) was very crystalline showing several significant peaks in the spectrum, in comparison with the original glass (Fig. 1a) which showed a very broad peak indicating the amorphous nature of the glass. Figure 1c–f shows that the degree of crystallization of the glass decreased as the sintering temperature decreased. By visual inspection, it was observed that the foams obtained at sintering temperatures of 600 and 575 °C were darker in color than those obtained at higher sintering temperatures.

#### TGA

**Fig. 2:**
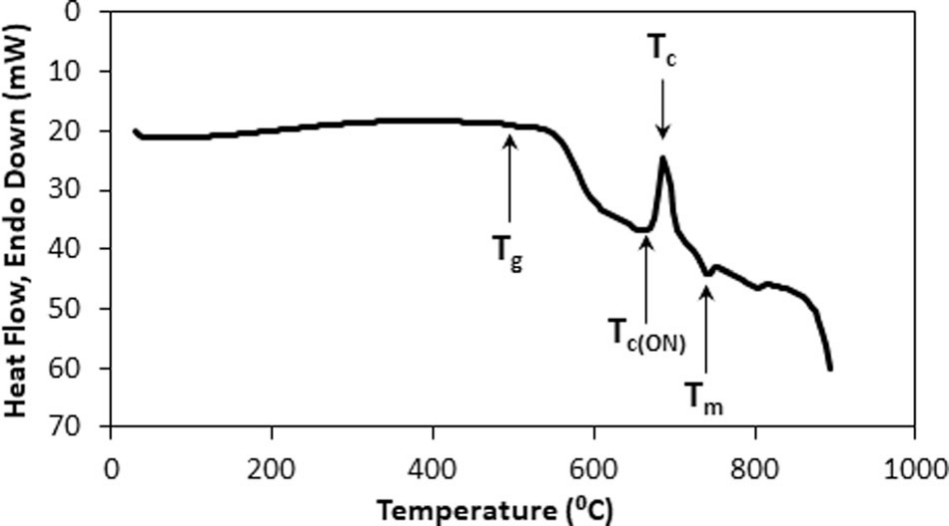
DTA of milled phosphate glass

Figure 2 shows the DTA curve obtained from the TGA data for the original glass before the foaming procedure was carried out. The DTA graph revealed a single sharp crystallization peak (T_c_) at 660 °C and the melting point (T_m_) at 740 °C. The onset of crystallization (T_c(ON)_) started at 640 °C and (T_g_) was observed to be at ~500 °C.

#### FTIR

**Fig. 3:**
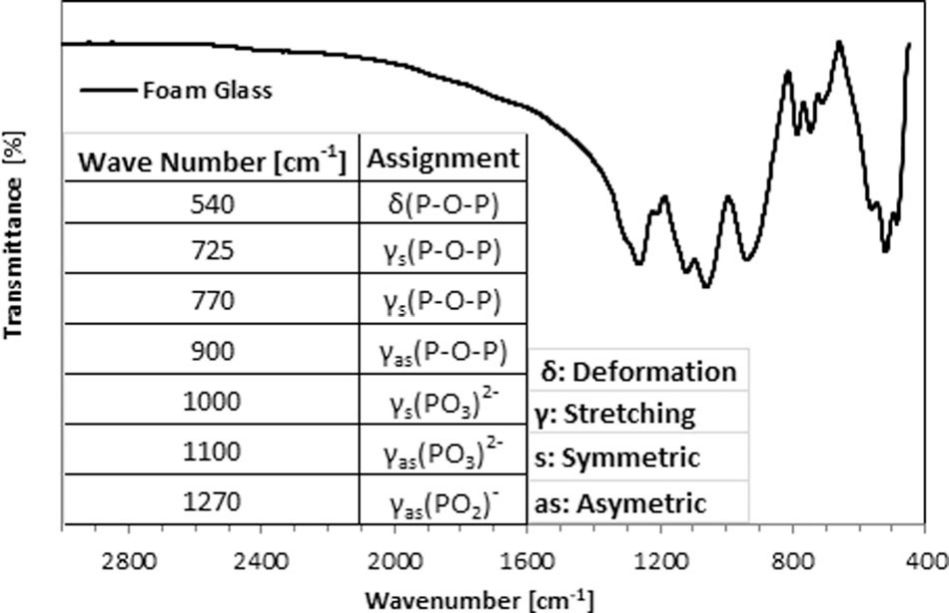
FTIR spectra of glass after sintering

Figure 3 shows the FTIR spectrum of glass after sintering in the fingerprint region (1500 to 400 cm^−1^). The peaks at 1270 and 1100 cm^−1^ were assigned to the asymmetric stretching of PO_2_^−^ (γ_as_(PO_2_^−^)) and PO_3_^2−^ (γ_as_(PO_3_^2−^)) groups. The peak at 900 cm^−1^ was assigned to the asymmetric stretching of P–O–P chains (γ_as_(P–O–P)). The peak with value located from 770 cm^−1^ to 725 cm^−1^ was assigned to the symmetric stretching of P–O–P chains (γ_s_(P–O–P)), peak value 1000 cm^−1^was assigned to symmetric stretching of γ_as_(PO_3_^2−^). The peak at 540 cm^−1^ was assigned to deformation of δ (P–O–P). Only peaks relating to phosphate groups were observed in the FTIR spectra. Importantly, no peak corresponding to C–H bond (region of 2900 cm^−1^) [36] and C=O bond (region of 1653 cm^−1^) [37] was observed in the glass after sintering, suggesting no presence of organic matter in the sintered glass sample.

#### SEM

**Fig. 4:**
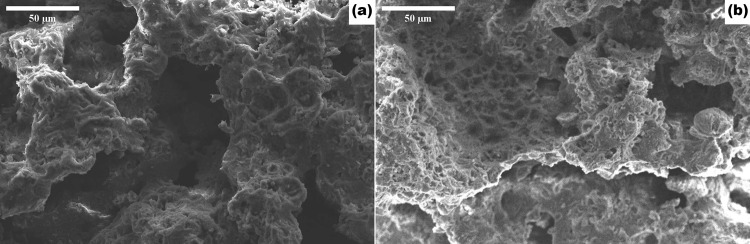
Representative SEM of 210–420 µm (**a**) and 420–850 µm (**b**) mesh fraction of PPG morsels. Scale bar = 50 µm

From Fig. 4, the surface was observed to be rough and the pores appeared to be interconnected, indicating high surface area and highly porous morphology. It was also observed that the porous nature of the morsels was not destroyed after milling and sieving of the particles to sizes in the range of 210–420 µm (Fig. 4a) and 420–850 µm (Fig. 4b) mesh.

#### PSD

**Fig. 5:**
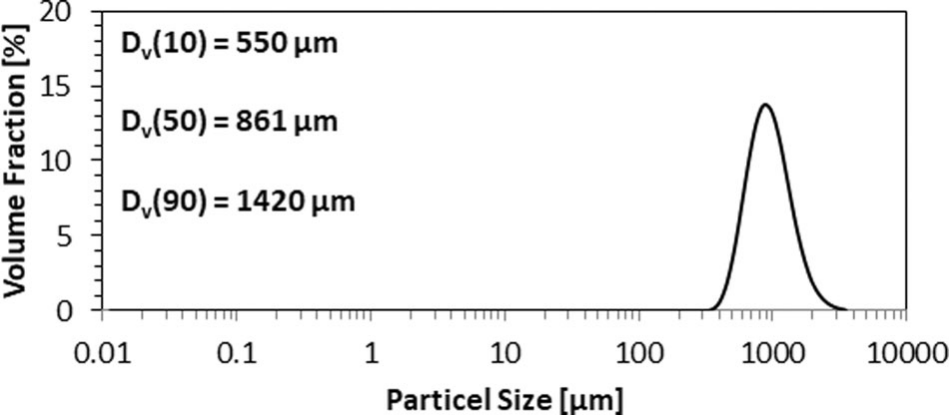
PSD of PPG morsels: 420–850 µm mesh fraction

Figure 5 shows the particle size distributions of the PPG morsels of 420–850 µm mesh fraction. It was observed that the particle size ranged from 400 µm to 2000 µm and that the average particle size is ~1000 µm. The D_v_ (10), D_v_ (50), and D_v_ (90) values were observed to be 550 µm, 861 µm, and 1420 µm respectively.

#### Mercury porosimetry

**Fig. 6:**
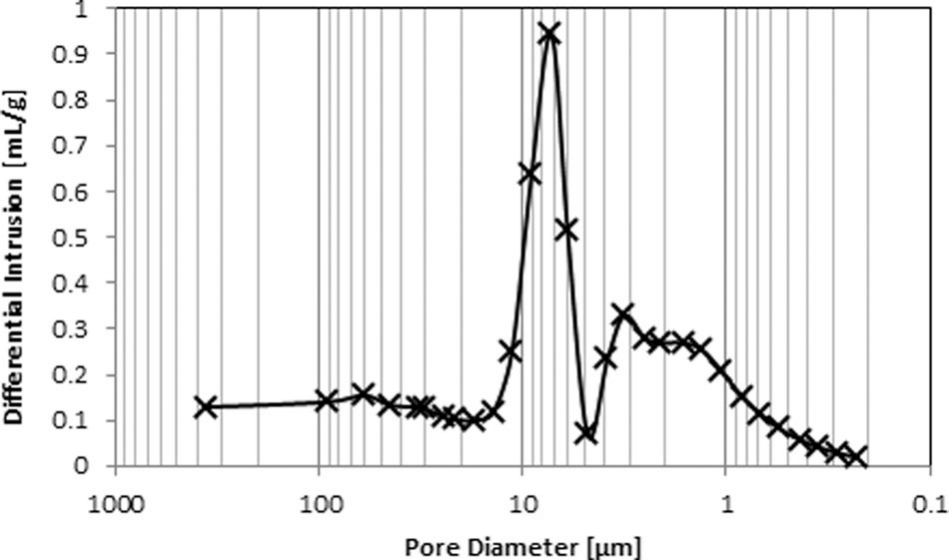
Mercury intrusion curves of PPG measured by mercury porosimetry: pore diameter distribution

Figure 6 shows the mercury intrusion profile. The intrusion profile shows a small mercury penetration into pores between 100 and 20 µm. This mercury penetration is attributed to the inter-particle pores [38]. Significant mercury penetration can be observed between 20 and 1 µm, indicating the presence of intra-particle pores. The average pore diameter (volume) is 2.8 µm and the average porosity is 58.45%.

#### Bulk density

It was observed that the bulk density of glass was 1.379 g/mL and that of the glass morsel (PPG) was 0.487 g/mL. This indicated the highly porous nature of the glass morsels having the same chemical composition as the original glass but with a porous 3D structure.

#### Degradation study

**Fig. 7:**
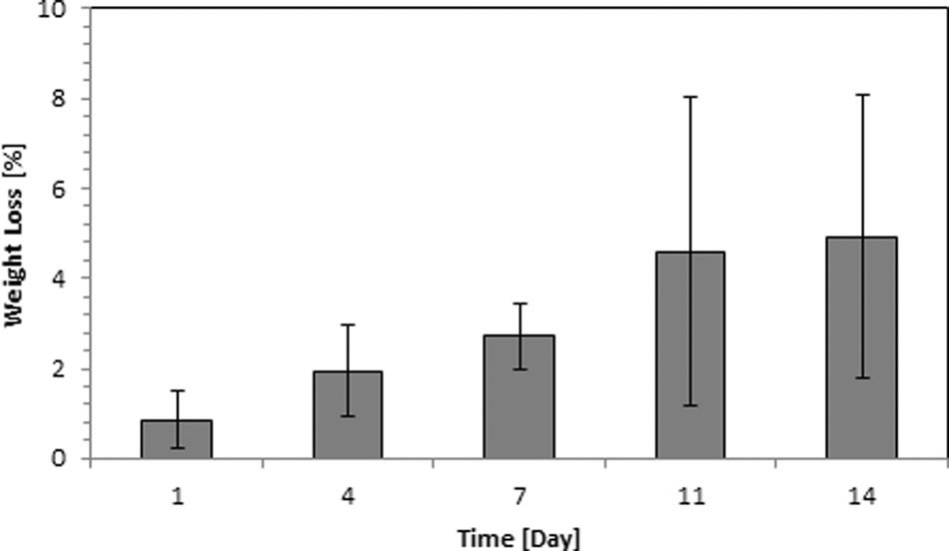
Degradation of PPG morsels presented as percent cumulative weight loss over a period of time

Figure 7 shows the percentage cumulative weight loss of PPG morsels over time points: Day 1, 4, 7, 11, 14. The total weight loss after 14 days was ~5%.

### In vitro studies

#### Cytotoxicity

Cell viability of more than 70% was observed in all dilutions of the extracted samples viz. 100, 50, 25, 12.5 and 6.25% when compared with the negative control (complete media). Based on the results, it was concluded that PPG is non-cytotoxic when tested with undiluted and diluted extracted samples using the MTT cytotoxicity assay with L929 cell lines.

#### SEM

**Fig. 8:**
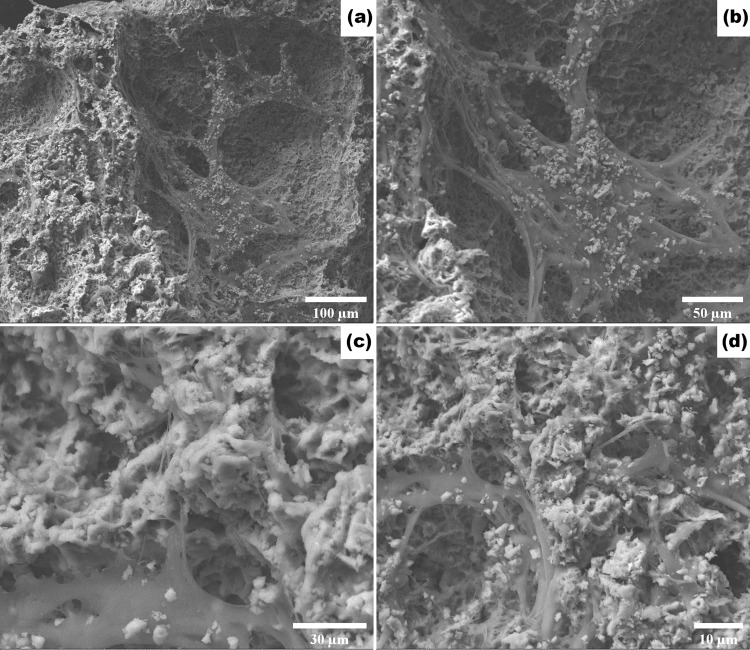
Representative scanning electron micrographs of DPSCs seeded on PPG morsels for 14 days. Scale bar for respective images are as follows: Image (**a**) 100 µm; (**b**) 50 µm & (**c**) 30 µm; (**d**) 10 µm

Figure 8 shows the cells and tissue formed on the surface of PPG morsels after 14 days of incubation. Almost all the cells exhibited a flattened morphology and were well-adherent to the rough surface of the morsels. In several cases, the cells were connected to each other by means of cellular processes. As seen in the images of second row, the cells also formed well-developed tissue structures in the pores of the morsels. This suggests that the internal surface of the pores is also used by the cells for proliferation.

#### Cell proliferation

**Fig. 9:**
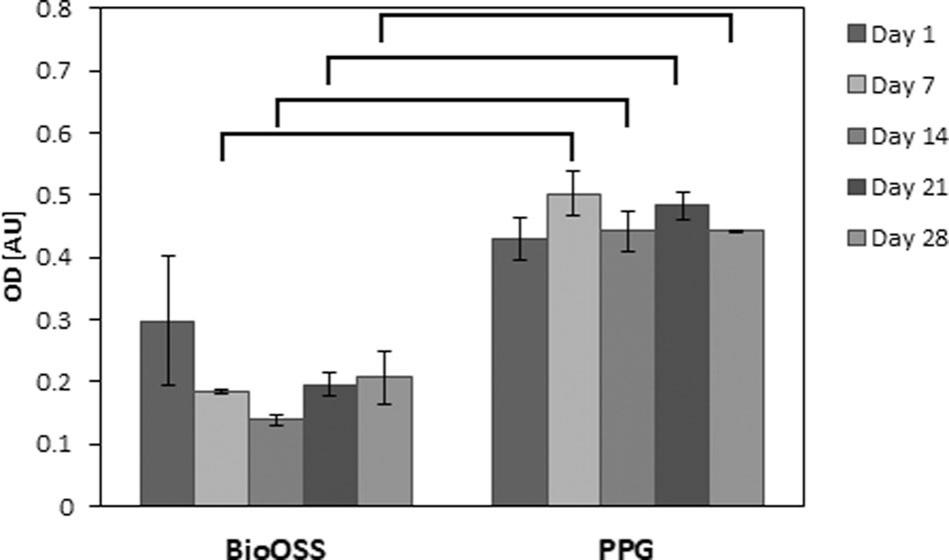
Comparative cell proliferation graph of PPG and XG

Figure 9 shows the results of the DPSC proliferation assay carried out over a period of 28 days on PPG compared with commercially available XG. The results revealed that the cells seeded in the tissue culture plastic plate grew normally. XG showed considerably less cell proliferation than PPG. Statistical analysis revealed significant differences (*p* < 0.02) between XG and PPG results of day 7, 14, 21 and 28 (Fig. 9). No significant difference was observed in day 1.

#### Confocal microscopy

**Fig. 10:**
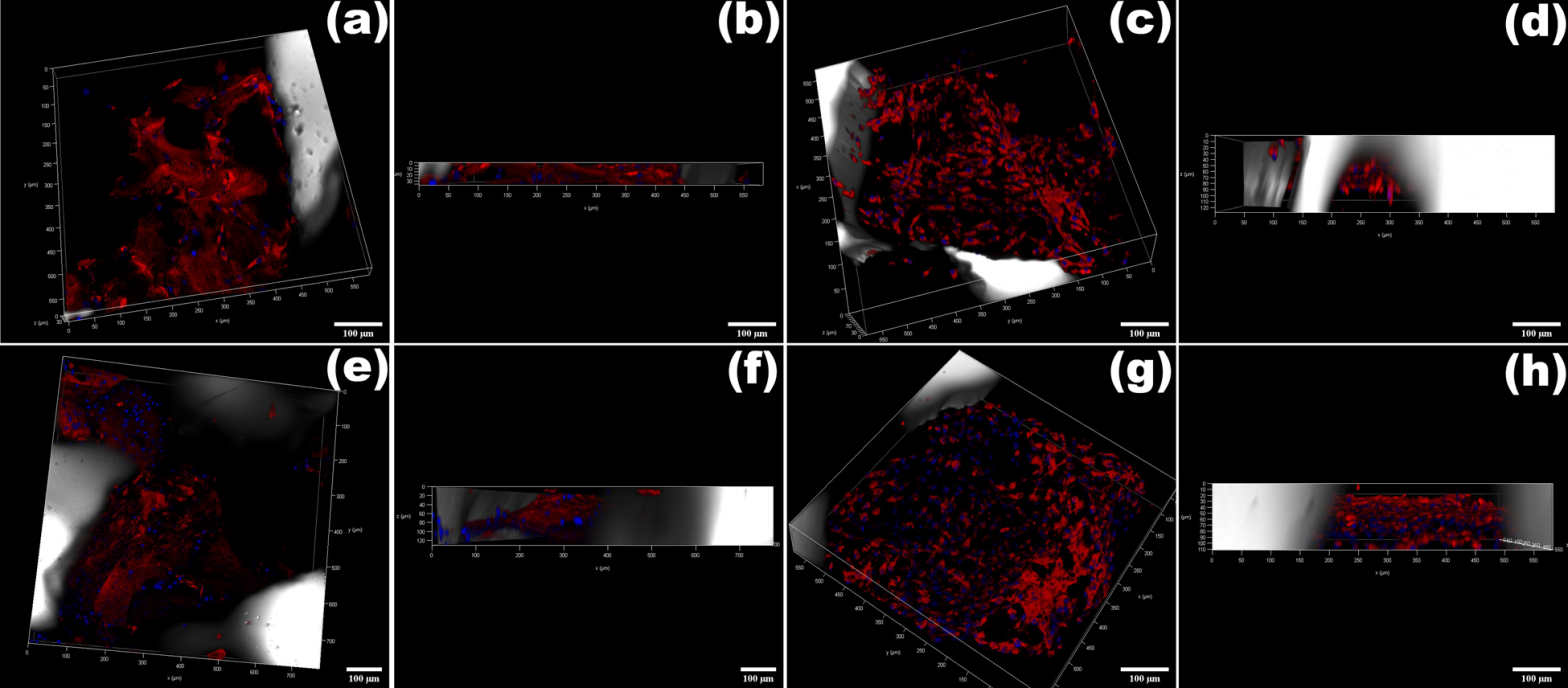
**a–d** Confocal laser scanning microscopy images of DPSCs attached to PPG and XG at 7 days post culture (scale bar- 100 µm); **e–h** Confocal laser scanning microscopy images of DPSCs attached to PPG and XG at 14 days post culture (scale bar- 100 µm)

The Methyl ester-Hoechst stained cells, which stained intracellular membrane red and nuclei blue, showed cell attachment along the biomaterial surface, implying that both PPG and XG have good cell adhesion characteristics. Figure 10a and c represent the transmittance light 3D image of biomaterial with the interaction of the cells in the XG and PPG respectively at 7 days post culture. Figure 10b and d represent the depth of the cells in XG and PPG respectively. From the images, it can be observed that the depth penetration of the cells was more in case of PPG (120 µm deep) as compared with XG (30 µm deep). Similarly, Fig. 10e and g represent 3D image and f and h represent depth of the cells penetrated in XG and PPG respectively at 14 days post culture. At 14 days post culture, the depth of cells penetrated for both XG and PPG was 120 µm. In case of XG, over a period of 7–14 days the cells penetrated deeper inside and in case of PPG the depth penetration was same i.e., 120 µm deep over a period of 7–14 days but a significant increase in number of cells was observed with respect to 7 and 14 days post culture. Also more number of cells was observed on PPG than on XG at 14 days post culture.

## Discussion

One of the major advantages of phosphate glasses over other glass systems, such as silicate and borate glasses, is that a very large proportion of the glass network is composed of components found naturally in bone (Ca, Na, P). Each component has a role in osteogenesis and eventually helps in bone regeneration [20, 39–41]. The glass undergoes a time-dependent kinetic modification of the available surface when implanted in living tissue, resulting in an osteoconductive and osteostimulative effect on the host bone tissue [11, 42–45].

As mentioned previously, it was hypothesized that incorporating porosity in the glass would significantly enhance its functional characteristics as a bone graft [38]. During foam making, methacrylamide was used as the monomer and N, N′ methylene bisacrylamide was used as the cross linker. To allow foaming, Triton X100, a surfactant was added followed by vigorous agitation. Ammonium polyacrylate was added as it plays the role of disperser and stabilizer; it helps in dispersion of glass particles in the solution and helps to stabilize the air bubbles in the liquid slurry. Addition of ammonium persulphate to the foam initiates the polymerization reaction of acrylamide and bisacrylamide by creating oxygen-free radicals. The reaction is completed by a base-catalyzed mechanism by the catalyst N, N, N′, N′- Tetramethyl ethylenediamine. The combined reaction of the monomer, cross linker and free radicals forms a polymer network. At this point, the glass particles are embedded in the polymer of the foam. The foam was then sintered wherein the high temperature calcines the polymer and the organic matter and the glass particles fuse together forming a porous foam of only glass [46].

It is crucial for the PPG morsels thus obtained to retain the bioactivity of the original glass. For this purpose, the amorphous nature of the original glass (i.e., the absence of crystalline phases in the glass) must be retained to the greatest possible extent. The glass becomes less bioactive with increase in crystalline phases as these phases resists ion exchange between the aqueous solution and the glass [47]. The sintering temperature should be within the window of T_g_ and the onset of T_c(ON)_ which ensures that the glass sinters without crystallization [48]. Though within the sintering window (500–640 °C), glass foams sintered at 600 and 575 °C appeared to be darker in color than the other samples sintered at higher temperatures of 625 and 650 °C, possibly due to incomplete calcination of the organic matter. In order to avoid being very close to the onset of the crystallization temperature, the sintering temperature of 625 °C was considered appropriate to largely retain the amorphous nature of the glass.

During the procedure of glass foam/morsels preparation, sintering is employed as it is necessary to remove the organic matter of the polymer and other unreacted organic ingredients used during foam preparation. The ingredients used in foam making such as methacrylamide, TEMED or ammonium polyacrylate may lead to toxicity even if they are present in trace amounts [49]. In addition, if during sintering, the organic matter is not calcined completely, it may hinder fusing of glass particles and block the pores, thereby reducing the porosity of the biomaterial. FTIR was employed to ascertain the absence of organic matter in the PPG morsels. A peak analysis in the fingerprint region is very complicated due to the large number of different vibrations that occur here which frequently overlap each other. These include single bond stretches such as C–C, C–O and C–X (where X–halide) and a wide variety of bending vibrations. However, a key observation was the absence of any peak corresponding to the C–H bond (region of 2900 cm^−1^) and C=O bond (region of 1653 cm^−1^) [36] in the glass after sintering, thereby confirming the absence of organic matter (Fig. 3).

According to the available clinical literature in dentistry [50, 51], the morsel size should be in the range of 200–800 µm for dental and maxillofacial applications. There are commercially available synthetic as well as non-synthetic bone grafts including xenografts of the above-mentioned size range particles. Therefore, before proceeding, SEM was performed to find out whether the morsels retain their porosity within the defined average particle size range; the SEM results confirmed the retention of porosity at the required particle sizes. For further use, the morsel size in the range of 420–850 µm mesh size was used. The PSD of this fraction was determined (Fig. 5).

Porosity being the novel feature of PPG, pore size distribution and porosity was determined using mercury intrusion porosimetry. These two parameters not only predict the area available for cell attachment but also provide important information about the potential for nutrient exchange to occur within the material structure. The incorporation of porosity in a material increases its total surface area as well as the pore volume. Larger surface area allows greater amounts of blood cells, proteins, and growth factors to be absorbed onto the morsels [52]. Although the average pore size as determined by mercury intrusion porosimetry is ~8 µm, the number of pores in the size range of 20–5 µm is high as observed from the pore size distribution curve (Fig. 6). These pore sizes are similar to the size of DPSCs and therefore suitable for their penetration and proliferation. The smaller pores are necessary to form blood vessels and induce both bone growth and tissue reorganization around the graft material [35]. The increase in the internal surface area and volume as an effect of foaming was confirmed by bulk density results. A high bulk density reflects less or no porosity while low density suggests high pore volume as observed for the original glass and the morsels respectively.

As known from the literature, degradation of phosphate glasses is highly controllable depending on various factors viz. mainly the metal oxides added to form quaternary structure; density; surface area and the local environment/solubilizing medium [53]. Here, in this study, a degradation study of PPG morsels was carried out to understand the trend of degradation of porous morsels. An initial linear trend was observed until Day 11 and later the degradation slowed (Fig. 7). Previous studies indicated that the degradation might take place in two different phases, which is most commonly observed in glasses with low Na_2_O content, similar to the glass used in the preparation of PPG morsels [53]. The two different phases involve (1) an initial fast process wherein ion exchange takes place where alkali ions like Na+ are exchanged by H_3_O+ ions, which leads to a mostly linear trend with respect to time, followed by (2) a slower process in which gradual bulk degradation takes place, involving breakdown of the remaining structure. This process is mainly driven by the interaction between phosphate chains and modifying oxides [44]. The data of this study can be related with the cell proliferation study (Fig. 9) where an increasing trend is observed from Day 1 to Day 7, which can be related to fast degradation and later a decline in proliferation was observed.

As explained by Patil et al, for any novel biomaterial, synthesis, characterization, in vitro and ex vivo studies shall be evaluated to understand its application in a particular field [54, 55]. The in- vitro evaluation includes studies like cytotoxicity, cell proliferation, cell differentiation etc. The biological evaluation starts with the assessment of cytotoxic effects. Cytotoxicity is the toxicity of the biomaterial to the cells. As per our results, a negative result indicated that the material is free of cytotoxic substances that may adversely affect cell viability [56]. The interaction of progenitor cells such as the DPSCs with the biomaterial is important to understand the possible in vivo behavior of bone grafts. Therefore, the interaction of DPSCs with PPG morsels was evaluated by SEM. The results revealed significant proliferation of DPSCs on the rough surface of PPG, indicating strong influence of the material surface morphology, porosity, and ion release on cell growth, proliferation, and adhesion [15, 53, 57].

In accordance with the results of the SEM study (Fig. 8), the cell proliferation assay results provided quantitative confirmation of the biocompatibility of PPG. It is worth mentioning here that XG was chosen for comparison instead of other available bone graft substitute types because it possesses a porous structure similar to PPG with a porosity of ~60% [58] and is demonstrated to exhibit osteoconductivity [8]. Although the bone regeneration abilities of XG and PPG are likely to be governed by different modes of action, the authors considered that the effects of both materials are nonetheless worth comparing at the in vitro level. Further comparison at the in vivo level will be provided in a future study.

Greater cell proliferation was observed on PPG than on XG at all the time points from day 7 onwards (Fig. 9). This is probably due to the combined effect of the inherent bioactive nature of the titanium phosphate glass material together with the porosity and higher surface area of PPG. The titanium phosphate glass used to produce PPG is known to be completely degradable and has a resorption profile that can easily be modified by subtle changes in chemical composition [9, 10, 15]. In previous comparative studies, it was observed that other synthetic biomaterials and porous calcium phosphate scaffolds showed a better degradation rate when compared with XG [59]. Although different opinions on the biodegradation of XG exist in the literature, the prevailing view is that XG can be resorbed and could be advantageous where bone resorption after augmentation should be prevented [17, 60].

To evaluate the cell interaction and penetration of DPSCs in PPG and XG, confocal microscopy was carried out at 7 days and 14 days post culture. The 3D stacked confocal images (Fig. 10a–h) depict the morphology of single XG and PPG particles and the interaction of DPSCs on these respective particles. Fluorescent cells were detected within the biomaterials at different levels through the z-stack, suggesting their localization within the pores. As observed in the confocal images, at day 7 post culture, deeper cell penetration (120 µm deep, Fig. 10d) was observed for PPG than for XG. This may be possibly due to better access to the porous interconnected void structure of PPG. But at 14 days post culture, the cell penetration depth was roughly equal (120 µm deep, Fig. 10f and h) for both PPG as well as XG. The possible reason for this could be that in the case of PPG, no more access to the void space was left for the cells to penetrate deeper inside the material structure due to presence of pores smaller than the cells. However, this would not hamper the ion exchange or adversely affect the cell proliferation as the ion release would progressively facilitate cell-to-cell interaction via cell signaling and also allow the already attached cells to proliferate within the particular local environment. Hence, when confocal data (Fig. 10) is compared with the proliferation results (Fig. 9) where the difference between XG and PPG cell proliferation was statistically significant (*p* < 0.02), it can be inferred that due to more porosity (more surface area and more void space), more number of cells could attach to the morsels, and eventually via higher levels of ion release and cell signaling, cells on PPG could undergo greater proliferation. Visual inspection revealed that after days 7 and 14, more cells were observed in the PPG images than in the XG images (Fig. 10a, c, e and g). This observation, although qualitative, is in agreement with the cell proliferation results which revealed greater cell numbers for PPG than for XG at both 7-day and 14-day time points.

## Conclusion

Novel porous phosphate glass materials were synthesized and their material properties and biocompatibility were evaluated. XRD and TGA data confirmed that the sintering temperature for the morsels should be 625 °C so that the morsels retain the amorphous nature of the original glass material to the maximum extent possible. The FTIR data proved the absence of organic matter, indicating that the sintering program is appropriate. The particle size was ~1000 µm for the mesh fraction of 420–850 µm PPG morsels. The SEM images for morsels suggest that the morsels retained their porosity even after undergoing the processing steps of milling and sieving. The results of mercury intrusion porosimetry revealed the porous nature of PPG having interconnected pore structures. Degradation studies showed increasing degradation of PPG morsels over a period of time. The PPG morsels were non-cytotoxic and the proliferation of DPSCs was greater on PPG than on the commercially available XG product. SEM and confocal imaging showed favorable interaction of DPSCs with the biomaterial on the external surface as well as within the bulk material. Confocal imaging also suggested superior cell interaction and proliferation in case of PPG in comparison with XG. Future in vitro studies are proposed for evaluating the ability of PPG to stimulate DPSC differentiation into osteogenic cell lineage. In addition, in vivo studies on different animal models will be conducted to further validate the biocompatibility of PPG at the preclinical level.
